# VR‐based Physical Activity Improves Cognition in Frail Nursing Home Residents with Diagnosed or Suspected Dementia in Hong Kong

**DOI:** 10.1002/alz70858_102598

**Published:** 2025-12-25

**Authors:** Mandi TANG, Ge Lin Kan

**Affiliations:** ^1^ Hong Kong University of Science and Technology (Guangzhou), Guangzhou, Guangdong, China

## Abstract

**Background:**

Physical activity is a promising clinical intervention for slowing the progression of Alzheimer's disease. But it understudied in people with frailty.

**Method:**

A rehabilitation bike with immersive familiar VR streets in Hong Kong was provided for this physical activity intervention program. A total of 21 frail nursing home residents (age: mean = 81.17, sd = 9.06) with diagnosed or suspected dementia participated in a 12‐week physical activity (PA) intervention program to verify if PA can improve cognition. All participants continued their standard care if any. Cognition (Montreal Cognitive Assessment/MoCA) and physical function (Activities of Daily Living/ADL‐BI) were assessed before and after the intervention program.

**Result:**

MoCA scores improved significantly following the 12‐week physical activity intervention, increasing from a mean of 14.39 (SD 5.59) to 17.61 (SD 7.41), with a mean difference of 3.22 (*p* <0.001).

**Conclusion:**

VR‐based physical activity is an effective intervention for improving cognition in frail nursing home residents with diagnosed or suspected dementia. This results demonstrated the potential to slow the progression of AD.

